# Expression analysis of RNA sequencing data from human neural and glial cell lines depends on technical replication and normalization methods

**DOI:** 10.1186/s12859-018-2382-0

**Published:** 2018-11-20

**Authors:** V. Bleu Knight, Elba E. Serrano

**Affiliations:** Department of Biology, New Mexico State University, Las Cruces, NM USA

**Keywords:** RNA Seq, Human neural stem cells, Human astrocytes, Glia, Normalization, Next generation sequencing, Experimental design, Principal variance component analysis

## Abstract

**Background:**

The potential for astrocyte participation in central nervous system recovery is highlighted by in vitro experiments demonstrating their capacity to transdifferentiate into neurons. Understanding astrocyte plasticity could be advanced by comparing astrocytes with stem cells. RNA sequencing (RNA-seq) is ideal for comparing differences across cell types. However, this novel multi-stage process has the potential to introduce unwanted technical variation at several points in the experimental workflow. Quantitative understanding of the contribution of experimental parameters to technical variation would facilitate the design of robust RNA-Seq experiments.

**Results:**

RNA-Seq was used to achieve biological and technical objectives. The biological aspect compared gene expression between normal human fetal-derived astrocytes and human neural stem cells cultured in identical conditions. When differential expression threshold criteria of |*log*_*2*_ *fold change|* > 2 were applied to the data, no significant differences were observed. The technical component quantified variation arising from particular steps in the research pathway, and compared the ability of different normalization methods to reduce unwanted variance. To facilitate this objective, a liberal false discovery rate of 10% and a |*log*_*2*_ *fold change|* > 0.5 were implemented for the differential expression threshold. Data were normalized with RPKM, TMM, and UQS methods using JMP Genomics. The contributions of key replicable experimental parameters (cell lot; library preparation; flow cell) to variance in the data were evaluated using principal variance component analysis. Our analysis showed that, although the variance for every parameter is strongly influenced by the normalization method, the largest contributor to technical variance was library preparation. The ability to detect differentially expressed genes was also affected by normalization; differences were only detected in non-normalized and TMM-normalized data.

**Conclusions:**

The similarity in gene expression between astrocytes and neural stem cells supports the potential for astrocytic transdifferentiation into neurons, and emphasizes the need to evaluate the therapeutic potential of astrocytes for central nervous system damage. The choice of normalization method influences the contributions to experimental variance as well as the outcomes of differential expression analysis. However irrespective of normalization method, our findings illustrate that library preparation contributed the largest component of technical variance.

**Electronic supplementary material:**

The online version of this article (10.1186/s12859-018-2382-0) contains supplementary material, which is available to authorized users.

## Background

RNA “sequencing by synthesis” (RNA-Seq) emerged over a decade ago as part of a suite of next generation analytical methods that enable high throughput interrogation of genomes and transcriptomes. RNA-Seq is becoming the method of choice for gene expression analyses due to technological advances that have increased genome coverage and reduced sequencing costs. RNA-Seq data acquisition mandates a substantial investment from the investigator; therefore, it is important to understand choices that may introduce bias or decrease the quality of the data. RNA-Seq poses particular challenges for researchers because standardized best practices have yet to be universally adopted [1]. Although next generation sequencing has fueled rapid advances in data generation and statistical analyses, technical procedures in the RNA-Seq workflow have commanded relatively less scrutiny (reviewed in [2]).

RNA-Seq has proven especially informative for the detection of genes that are differentially expressed during biological processes such as organism development and disease progression. The identification of differentially expressed genes is achieved with a multi-step workflow, and bias can be introduced in both the data-generation and data-analysis phases. The selection of appropriate normalization and data analysis methods have received considerable attention, and statistical algorithms that are specifically designed to address RNA-Seq data postprocessing and evaluation continue to evolve [3–9]. However, experiments have shown that the results of RNA-Seq experiments can also be affected by technical aspects of data generation, including the quality and amount of RNA [10, 11] and library preparation [12–14]. These experimental findings illustrate that the RNA-Seq outcomes can be confounded by the introduction of technical variation as part of sample processing during different phases of data acquisition and analysis. The quantification of experimental variance that can be introduced by different stages in the workflow would be useful because of the difficulty and expense that are involved in RNA-Seq data acquisition, and to address experimental objectives for reproducibility.

In the current study, we quantify the variation introduced during the experimental workflow, with the long term goal of increasing the fidelity of ongoing experiments with human cell lines derived from brain tissue [15, 16]. To this end we collected RNA-Seq data from two distinct neural cell lines, the federally approved H9-derived human neural stem cell line (hNSC) and normal human fetal-derived astrocytes (NHA). The motivation for comparing human fetal-derived astrocytes with human neural stem cells is the result of a body of literature that demonstrates the diverse plastic capacity of astrocytes. In previous work, we show that normal human fetal-derived astrocytes assume morphological and transcriptomic properties that are typically ascribed to neurons [16]. Others illustrate that, in many regions of the brain, astrocytes regain the capacity to proliferate within the astroglial lineage after brain injury (reviewed in [17]). Astrocytic plasticity is also illustrated by the finding that reactive astrocytes assume characteristics of neural stem cells [18, 19]. Moreover, in vitro analyses demonstrate the capacity of reactive astrocytes to proliferate outside of their lineage, and differentiate into neurons [17–20]. These findings illustrate the potential for astrocytes to participate in neuronal regeneration after brain injury, and demonstrate the need for increased research efforts in this arena. Thus, this transcriptomic analysis was undertaken with two primary objectives: (1) to investigate potential parallels in the neuroplastic capacities of normal human fetal-derived astrocytes and human neural stem cells, and (2) to assess variation imparted in the experimental workflow by technical replicates for RNA isolation, library preparation, and flow cell sequencing.

## Methods

### RNA sequencing

RNA-seq data can be confounded by the introduction of unwanted statistical variance at any procedural step of the data generation and data analysis procedures in RNA-Seq experiments (Table 1). Variation in RNA-seq data is typically regarded as random effects. Mixed models are implemented in statistics to fit experimental designs that include both fixed and random effects. The variance of each random effect is known as a variance component [21]. This study evaluates the potential variance contributions of steps 1, 3, 4, and 7 of the RNA-seq workflow (Table 1). For details regarding how steps in the RNA-seq workflow contribute to variance, the reader is directed to the thorough explanations provided in the references listed in Table 1.

**Table 1:** Steps in the RNA-Seq workflow and potential contributions to experimental variance. At each step in an RNA-Seq experiment, different parameters can introduce unwanted variance that obscure the results of gene expression analyses. Detailed discussions of these parameters are given by the sources in the References column

Step in RNA-Seq Workflow	Potential Contributions to Variance	Reference(s)
1. Experimental Design	number of replicate samples; genetic background of samples	[9]
2. RNA Isolation	RNA integrity number (RIN) value (RNA quality); isolation method	[1, 34]
3. Library Preparation	initial quantity of RNA template; RNA processing: polyA^+^ (mRNA enrichment), rRNA^−^ (rRNA depletion); preliminary amplification steps	[1, 34]
4. Sequencing	sequencing platform; depth of coverage; software for base-calls	[42]
5. Preprocessing	trimming adapter sequences and/ or low quality reads	[1]
6. Mapping	quality of reference genome, stringency	[1, 34]
7. Normalization	method	[8, 35, 37]
8. Statistical Analysis	method; stringency	[1, 7]

### Cell culture

#### Human neural stem cells

Gibco® H9 hESC-Derived Human Neural Stem Cells (hNSC; ThermoFisher Scientific, N7800100) were cultured in accordance with previously described protocols [22]. Briefly, the manufacturer’s specifications for hNSC were followed in order to culture two different cell lots (lot A, #1402001; lot B; #1408001). 2 mL StemPro neural supplement (Gibco®, A10508), 2 μg EGF (Gibco®, PHG0314), 2 μg bFGF (Gibco®, PH60024), and 1 mL Glutamax (Gibco®, 35,050–061) were combined with 97 mL Knockout DMEM/F-12 (Gibco®, 12,660–012) and filter sterilized with a 0.2 μm porous membrane to prepare 100 ml of complete hNSC serum free media, which were stored in 10 mL aliquots.

Cells were thawed, resuspended in complete hNSC serum free media, and centrifuged. The supernatant containing cryoprotectant was removed before resuspending in complete hNSC serum free media and transferring cells (passage 0) to T-25 flasks (one flask per ampule) coated with CellStart (Gibco®, A10142). Media were replenished following every 48 h of incubation at 37 °C, 5% CO_2_. When cultures were ~ 80% confluent, they were rinsed in DPBS (without calcium or magnesium) and partially digested with 2 mL of 37 °C StemPRO Accutase for subculturing. When detachment was observed under the microscope, cells were transferred with 9 mL of media to tubes for centrifugation at 210 G for 5 min. Supernatant was removed, cells were triturated in prewarmed media and transferred to T-25 flasks coated with CellStart.

#### Normal human fetal-derived astrocytes

Normal human fetal-derived astrocytes (NHA; Lonza, CC-2565) from two donor lots (lot A, #0000412568; lot B, #0000402839) were cultured according to previously established protocols [16, 22]. Vials of cells obtained from the vendor were thawed and cultured in T-25 flasks (passage 0) with media changes following every 48 h of incubation at 37 °C, 5% CO_2_. At ~ 80% confluence (day 5) cells were subcultured by partial digestion and plated in vessels recommended for hNSCs (T-25 flasks coated with CellStart; passage 1). 48 h after the first passage, media were changed to complete hNSC serum free media. NHA and hNSC were cultured in parallel conditions after this point.

#### Spontaneous differentiation

The second passages of lot A and lot B from NHA and hNSC were subcultured by partial digestion as described above, and cultured according to the manufacturer’s specifications for spontaneous differentiation as described previously [22]. Briefly, cells were titered using a hemocytometer and plated in T-25 flasks coated with poly-L-ornithine (Sigma P3655) and laminin (ThermoFisher, 23,017,015) at a density of 2500 cells/cm^2^ in complete hNSC serum free media. After 24 h, media were replenished with 97% Knockout DMEM/F-12, 1% Glutamax, and 2% StemPro neural supplement. Every 48 h, 75% of the media were replenished with 97% Knockout DMEM/F-12, 1% Glutamax, and 2% StemPro neural supplement while ensuring that cells were not exposed to air. After the 10-day differentiation protocol suggested by the manufacturer, images were captured and RNA was isolated as described in the following sections.

### Live cell imaging with phase contrast microscopy

Phase contrast images of living cells were acquired with Metavue image capture software (Molecular Devices) prior to RNA isolation. Images were captured with a Coolsnap HQ CCD camera (Photometrics) attached to the projection port of an inverted Nikon TE-2000 microscope.

### Sample preparation and sequencing

RNA was isolated as previously described [16, 22] using the instructions for the PureLink® RNA Mini Kit (Ambion). DNA was removed as previously described [16, 22] using the instructions for the DNA-free™ kit (Ambion). An Agilent 2100 Bioanalyzer was used to evaluate RNA quality. RNA samples (*n* = 4) with RIN Values greater than 8.9 were divided in half and submitted to the BioMicro Center at the Massachusetts Institute of Technology where two different libraries for each condition were prepared as follows (8 libraries total). 10 ng of total RNA was used as input for cDNA preparation with the SMART-Seq v3 Ultra Low Input RNA Kit for Sequencing (Clonetech) according to the manufacturer’s specifications. Fragmented samples were transferred to the SPRI-works for BioMicro Center adapter ligation, multiplex barcoding, size selection, and enrichment using BioMicro Center PCR primers. An AATI Fragment Analyzer™ (Advanced Analytical) was used to assess the libraries for fragment size and distribution. Multiplexed samples were sequenced twice according to the protocol for 150 base pair (bp) paired end (PE) reads on an Illumina NextSeq sequencer. Reads mapping to the forward and reverse strands were pooled because the libraries were not prepared with strand-specific protocols.

### Quality control, filtering, and alignment

Phred scores were assessed with FastQC, a quality control software program, to evaluate base call accuracy in accordance with previous methods [22, 23]. Reads with average minimum quality scores corresponding to 99% base call accuracy at every nucleotide position (Phred score > 20) were retained. The Burrows-Wheeler Aligner (BWA-MEM, v0.7.10) was used to align fastq files to the human genome (v hg 19). JMP Genomics (v 8.0, SAS Institute, Inc.) was used to import SAM files and summarize gene counts based on the UCSC human genome annotation (hg 19). A thresholding filter of a minimum raw read count greater than 10 per gene was applied to the data, yet no genes were removed following the application of this detection threshold. The evaluation of overall gene expression (instead of isoform-specific expression) facilitated the evaluation of data using standardized methods.

### Normalization

Read counts were normalized to account for varying lane sequencing depth and other potential technical effects as described previously [22]. JMP Genomics was used to log_2_ transform the sequence data and to normalize it using three different methods: (1) reads per kilobase of exon per million reads mapped (RPKM), (2) trimmed means of M component (TMM), (3) upper-quartile scaling (UQS). RPKM-normalized data is scaled by a factor that considers the both the library size and gene length. TMM-normalized data represents the average after removing the highest and lowest data points and does not consider library size. UQS-normalized data is adjusted based on the size of the library.

### Statistical analyses

In accordance with previous approaches, the data were fit to a Poisson model as part of the JMP Genomics ANOVA analysis [22]. Poisson models are established in the literature as representative distributions of the technical variation in RNA-seq [24, 25]. The step-up false discovery rate (FDR) method of Benjamini and Hochberg was used to adjust *p*-values for multiple comparisons in the statistical analysis undertaken with JMP Genomics [26]. The multiple comparison adjustment is important because of the large number of genes that are compared with RNA-seq data analysis [27]. The variance contributions from the fixed cell line variable and the random variables (library preparation, flow cell, cell lot; Table 2) were calculated with principal variance component analysis using JMP Genomics. Due to the similarities in the tissues of origin for these two cell lines, as well as the parallel culture conditions, we expected that a low number of genes would be differentially expressed. Therefore, potential differences in gene expression were evaluated using a false discovery rate of 10% and a |*log*_*2*_ *fold change*| value of 0.5. For a detailed description of the statistical analyses used in this study, refer to [28, 29] and the literature for JMP Genomics v 8.0.

**Table 2:** Experimental parameters contributing to variation in the study were assessed by analysis of eight forward and eight reverse RNA-Seq data files from each cell line (32 data files total)

Component	Number of Replicates	Type of Variation	Effect
Cell line	2 (NHA, hNSC)	Conditional	Fixed
Lot	2 per cell line	Technical	Random
Library preparation	2 per lot	Technical	Random
Flow Cell	2 per library	Technical	Random

### Responsible conduct and reproducibility

Our experimental design was influenced by the guidelines for preclinical research set forth by the NIH, as previously described [22]. The WA09 (H9) embryonic stem cell line served as the source of the hNSCs, and the NIH registry for human embryonic stem cells retains the information about the WA09 (H9) stem cell line (NIH approval number NIHhESC-10-0062). NHA were de-identified and produced by Lonza, who retains the record of donor consent. In accordance with the manufacturers’ specifications, both cell lines were used within 10 population doublings (3 passages). The human species origins of both cell lines were verified with RNA-Seq (see below). Although the review of experiments using de-identified, commercially available, human cell lines produced before 2015 is exempt from Institutional Review Board, the protocol described herein was approved by the NMSU Institutional Biosafety Committee (approval # 1401SE2F0103).

At the MIT BioMicro center, a single blind protocol was used to collect sequence data without prior knowledge of the nature of the biological samples. Groups were assigned to the sequence data by researchers at NMSU who assessed the outcomes. The standards set forth by the HUGO Gene Nomenclature Committee guided the use of official gene symbols in this manuscript.

## Results

### Live cell imaging with phase contrast microscopy

**Fig. 1:**
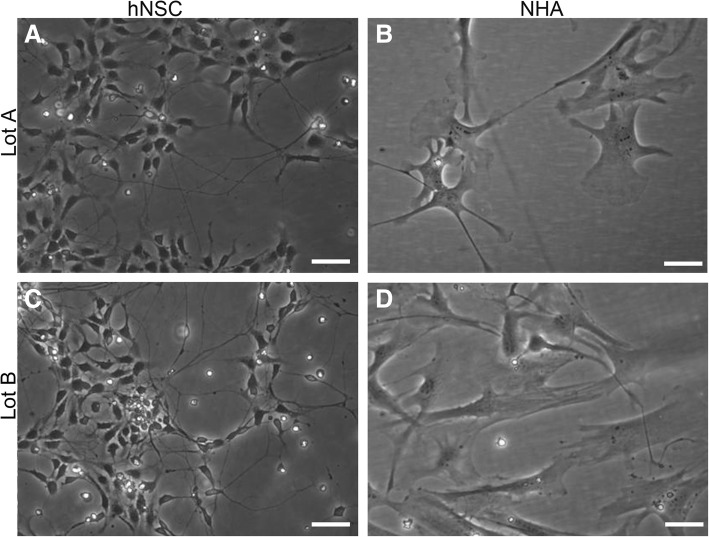
Neural stem cells (hNSC; **a**, **c**) and normal human astrocytes (NHA; **b**, **d**) were cultured under spontaneous differentiation conditions for 10 days. Live cells were imaged with phase contrast optics, prior to extraction of RNA for transcriptome analysis. Representative images are shown from both cell lines for two lots: Lot A (**a**, **b**); Lot B (**c**, **d**). Scale bar = 50 μm. Adapted by permission from Springer [22], Copyright 2017

Morphological differences between human neural stem cells and normal human fetal-derived astrocytes cultured in an identical spontaneous differentiation environment are visible in phase contrast images (Fig. 1). Human neural stem cells (Fig. 1a, c) appeared smaller in size and grew at a higher density than normal human fetal-derived astrocytes (Fig. 1b, d). The stellate morphology that is characteristic of astrocyte cultures was prevalent in normal human fetal-derived astrocyte cultures. The long processes, typical of neurons, were visible in both cell lines but were more ubiquitous in human neural stem cell cultures.

### Evaluation of sequence quality and read distribution

**Fig. 2:**
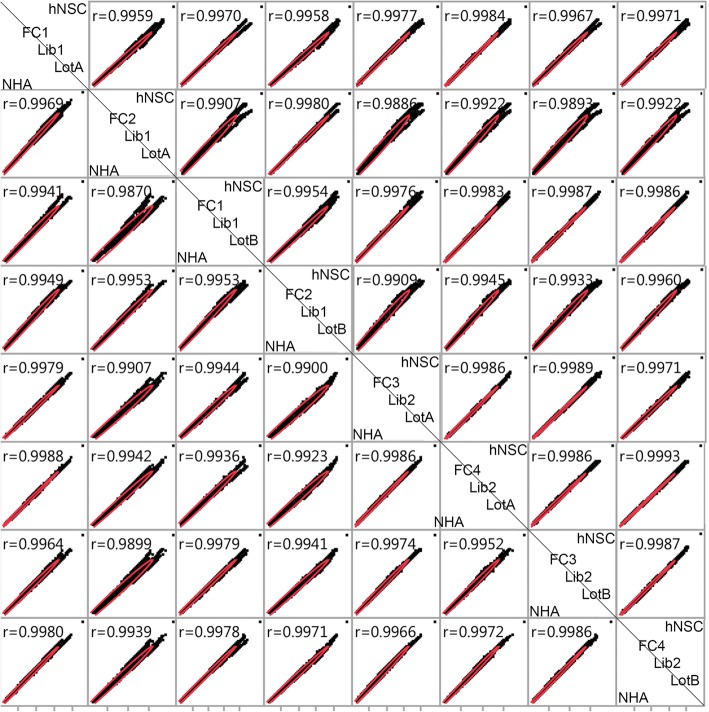
Scatterplot matrix illustrating correlation between technical replicates. Read counts for each gene are plotted against read counts from technical replicates for hNSC (top right of diagonal) and NHA (bottom left). Comparisons are made by flow cell (FC), library (Lib) and cell lot (Lot) for both cell lines. Correlation coefficients between each pair of samples are shown on the top left of each box

FastQC evaluation of RNA-Seq read quality revealed that in forward reads, the average Phred scores for all read positions met the criteria for 99% base call accuracy (Phred score > 20). In reverse reads, base pairs 140–150 dropped in quality from ~ 28 to ~ 14. Sample, library preparation and flow cell showed differences in the raw read count output (Table 3). The fourth flow cell provided a greater number of reads than the other flow cells (in some cases more than twice the amount). Data evaluation illustrated that reads were not preferentially distributed among smaller or larger genes. Moreover, the increase in reads from the fourth cell was distributed throughout the transcriptome. Multiple alignments per read (~ 70) were observed from all alignments, a finding that is consistent with other alignment data acquired from total RNA libraries constructed using the Clonetech SMART technology [30]. Scatterplot matrices were prepared to compare the distribution of data from technical replicates for each cell line (Fig. 2). The Pearson coefficient values (*r* > 0.98) indicate a strong positive correlation between all technical replicates for both cell lines.

**Table 3:** Sequencing reads counts from replicate samples. Two libraries (1,2) were prepared from RNA isolated from two lots of cells (A,B) from each cell line (NHA and hNSC). Data from two distinct flow cells was collected from each of the eight RNA-Seq libraries, for a total of 16 forward and 16 reverse data files

Cell line	Flow Cell	Lot	Library	Total Reads
hNSC	1	A	1	19,113,825
hNSC	1	B	1	19,551,997
hNSC	2	A	1	28,782,901
hNSC	2	B	1	29,327,349
hNSC	3	A	2	31,909,377
hNSC	3	B	2	30,372,982
hNSC	4	A	2	45,893,035
hNSC	4	B	2	44,421,228
NHA	1	A	1	19,293,695
NHA	1	B	1	14,893,476
NHA	2	A	1	28,848,603
NHA	2	B	1	22,180,928
NHA	3	A	2	34,703,198
NHA	3	B	2	34,057,549
NHA	4	A	2	50,551,021
NHA	4	B	2	49,729,736

### Normalization

**Fig. 3:**
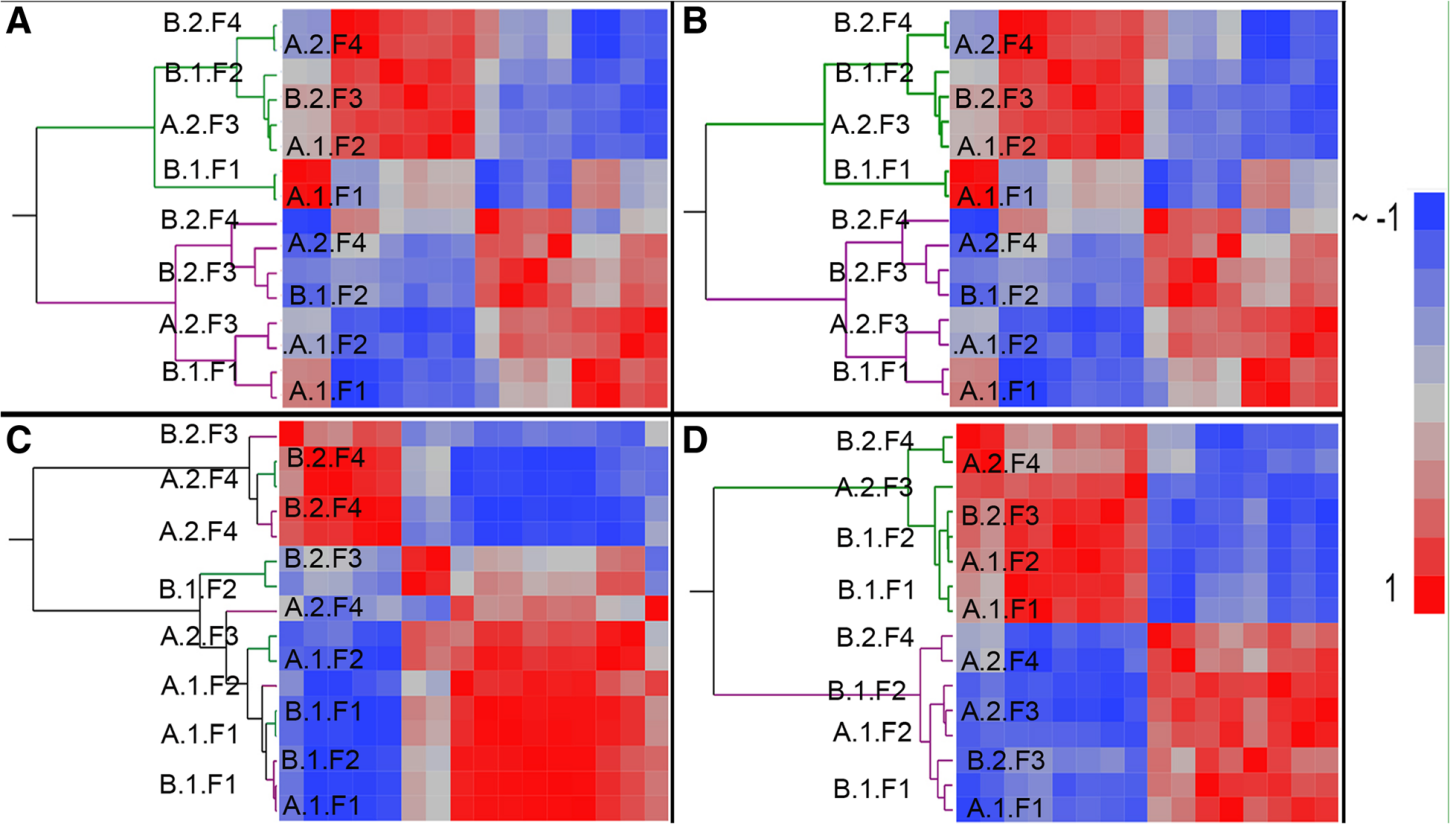
Heat maps depict Pearson correlation coefficients and hierarchical cluster analysis with the Ward method (Green, hNSC; Violet, NHA). Pearson correlation coefficients (r values; Blue ~ − 1, Red = 1) and clusters were computed for log2 transformed data (**a**), RPKM-normalized data (**b**), TMM normalized data (**c**) and UQS normalized data (**d**). The cell lot (A, B), library (1, 2), and flow cell (F1, F2, F3, F4) are identified with sample annotation in the order: lot.library.flow cell. Adapted by permission from Springer [22], Copyright 2017

**Fig. 4:**
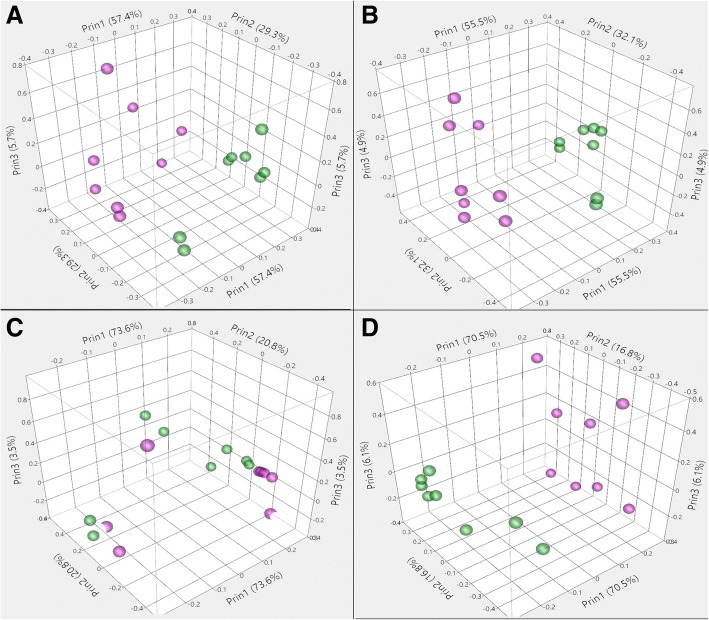
Principal component analyses (PCA) for log_2_ transformed data (**a**), RPKM-normalized data (**b**), TMM normalized data (**c**) and UQS normalized data (**d**) depict the effect of normalization on sample grouping by cell line (Green, hNSC; Violet, NHA)

Normalization methods were assessed by determining the reduction of variation between technical replicates and the presence of differentially expressed genes between the two cell lines. Heat maps of sample-to-sample correlation coefficients were clustered with the Ward method. The influence of normalization on the data is evident in the hierarchical cluster analysis (Fig. 3). Log_2_ transformed data, RPKM normalized data, and UQS normalized data cluster the samples based on cell line. In contrast TMM normalized data did not cluster samples based on any of the experimental parameters. The UQS normalized data produced the most coherent heat maps (Fig. 3d). Principal component analyses revealed a similar trend among the normalization methods (Fig. 4). In the PCA plots, samples were closely associated with other samples of the same cell line with data normalized using all methods except TMM (Fig. 4c).

### Lot, library preparation, and flow cell effects

**Fig. 5:**
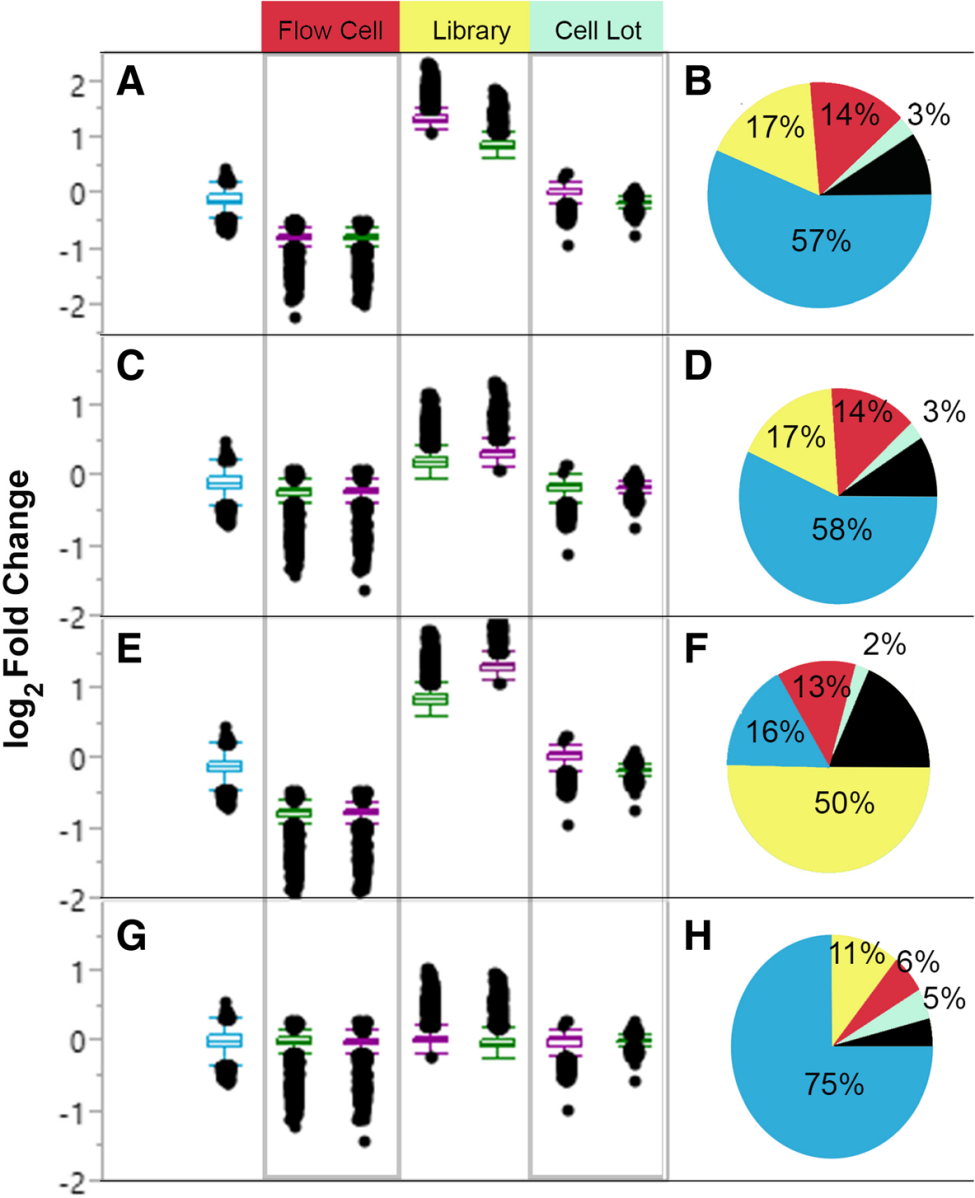
Conditional (cell line) and technical (library, lot, flow cell) contributions to variation between replicate samples. Box plots (**a**, **c**, **e**, **g**) show log2 fold-changes between technical replicates for both cell lines (hNSC, green; NHA, violet) and between cell lines (blue). Pie charts demonstrate that the contribution of different components to sample variance (**b**, **d**, **f**, **h**). Principal variance component analysis revealed the influence of cell line (blue), flow cell (red), library preparation (yellow), cell lot (light green), and re-sidual variance (black). The estimates are shown for log2 transformed data (**a, b**), RPKM normalized data (**c, d**), TMM normalized data (E,F) and UQS normalized data (G, H). Adapted by permission from Springer [22], Copyright 2017

The proportions of variance resulting from different experimental parameters were uncovered using principal variance component analysis with JMP Genomics (Table 2). The normalization method affected the variance proportions that were contributed by the experimental components (Fig. 5). Experimental variation between log_2_ transformed, non-normalized data and RPKM normalized data showed the smallest change of all normalization methods (< 2% different). In contrast, the proportion of variance contributed by library preparation was increased to over 50% in TMM normalized data, as compared to under 20% with other normalization methods. In TMM normalized data, the percentage of variance based on cell line decreased to 16% from over 50% with other normalization methods. The greatest contribution from cell line was seen in UQS normalization (75%) as compared to other methods (16%, TMM normalized; 57%, log_2_ transformed; 58% RPKM normalized). UQS normalization also resulted in the greatest contribution from the cell lot to the experimental variance (5%), which was almost double the contribution from lot in non-normalized data (3%) and in data normalized with other methods (3%, RPKM; 3%, TMM). The increased contributions from cell line and lot that were observed with UQS normalization were accompanied by decreased contributions from library preparation (11%) flow cell (6%), and residual (5%) variance as compared to other normalization techniques.

Differences between replicate cell lots, libraries, and flow cells were evaluated by looking at the *log*_*2*_ *fold change* values between replicate samples (Fig. 5). Regardless of normalization method, cell lot had the smallest distribution of *log*_*2*_ *fold change* values (Fig. 5). While replicate flow cells had larger *log*_*2*_ *fold change* values than cell lot, UQS normalization was found to reduce the *log*_*2*_ *fold change* values for flow cell by half (Fig. 5). Library preparation was found to have the largest distribution of larger *log*_*2*_ *fold change* values, regardless of normalization method (Fig. 5). We also found that TMM normalization increased the variance contributed by library preparation beyond the conditional variance that was observed between the two cell lines.

### Differential expression

We expected to observe similar gene expression patterns in the two cell lines selected for this study because both are of central nervous system lineage, and were cultured under identical conditions. Previous RNA-seq analyses by our laboratory have implemented differential expression criteria of |*log*_*2*_ *fold change|* > 2, *p*_*adj*_ < 0.1 [16]. When these restrictions are applied to the data, analysis uncovers no significant differential expression between normal human fetal-derived astrocytes and human neural stem cells cultured under identical conditions. The other goal of this study was to quantify technical variation that arises in the experimental workflow, and compare the effectiveness of different normalization methods in reducing unwanted variance. To facilitate this objective, we imposed a more liberal false discovery rate of 10% and a |*log*_*2*_ *fold change|* > 0.5 for the differential expression threshold criteria. This analysis revealed that the choice of normalization method affected our ability to detect significant differential gene expression between the two cell lines (Table 4). Even with these generous constraints, the analysis of differential expression resulted in no significant genes for RPKM normalized and UQS normalized data. In contrast, analysis of non-normalized data and TMM normalized data yielded 165 and 143 differentially expressed genes, respectively, after removal of duplicate genes. Table 4 reports the ten genes with the largest |*log*_*2*_ *fold change*| values for each normalization method. The maximum |*log*_*2*_ *fold change*| values detected by the four methods ranged from 0.41 (UQS normalized data) to 0.62 (non-normalized data).

**Table 4:** Genes with the largest fold change between hNSC and NHA cell lines (p-values)

^a^Log_2_Transformed	RPKM Normalized	^a^TMM Normalized	UQS Normalized
*PARD6G-AS1* (4.7 × 10^−3^)	*HNRNPU* (2.8 × 10^−10^)	*PARD6G-AS1* (4.7 × 10^− 3^)	ZNF496 (6.1 × 10^−7^)
*HNRNPU* (1.6 × 10^−7^)	ZNF496 (2.2 × 10^−8^)	*HNRNPU* (1.5 × 10^−7^)	*PARD6G-AS1* (1.9 × 10^−2^)
*RNF126* (4.8 × 10^−4^)	*PARD6G-AS1* (5.0 × 10^− 3^)	*RNF126* (4.9 × 10^− 4^)	*HNRNPU* (1.8 × 10^− 10^)
*OR4F17* (2.8 × 10^− 3^)	*RNF126* (2.0 × 10^− 4^)	*OR4F17* (2.8 × 10^− 3^)	ACTB (1.1 × 10^− 3^)
LINC01002 (2.3 × 10^− 3^)	*OR4F17* (2.6 × 10^− 3^)	LINC01002 (2.3 × 10^− 3^)	*RNF126* (1.6 × 10^− 3^)
LOC105376854 (2.7 × 10^− 4^)	LOC105376854 (9.6 × 10^− 5^)	LOC105376854 (2.7 × 10^− 4^)	*OR4F17* (1.2 × 10^− 2^)
HCN2 (5.8 × 10^− 4^)	MBD3 (6.1 × 10^− 6^)	HCN2 (5.9 × 10^− 4^)	LINC01002 (1.0 × 10^− 2^)
PRSS57 (5.3 × 10^− 4^)	ARHGAP45 (1.2 × 10^− 5^)	PRSS57 (5.5 × 10^− 4^)	ARHGAP45 (2.1 × 10^− 4^)
MBD3 (3.9 × 10^− 5^)	CIRBP-AS1 (8.0 × 10^− 6^)	MBD3 (4.1 × 10^− 5^)	ADAMTSL5 (6.5 × 10^− 5^)
ARID3A (2.4 × 10^− 4^)	ADAMTSL5 (8.3 × 10^− 6^)	CIRBP-AS1 (4.8 × 10^− 5^)	ARID3A (7.2 × 10^− 4^)

Significant^a^ differentially expressed genes were only uncovered following log_2_ transformation and TMM normalization (Benjamini-Hochberg FDR = 10%). Common genes shown in italics

Four of the top ten genes with the largest |*log*_*2*_ *fold change*| values between cell lines were identified by all normalization methods (*italicized*). Three genes were common to three methods, while six genes were identified by two methods, and one gene was exclusive to one method (Table 4). Nine of the ten genes with the largest |*log*_*2*_ *fold change*| values that were calculated with log_2_ transformed and TMM normalized data were identical. Seven of the top ten genes with the largest |*log*_*2*_ *fold change*| present in RPKM normalized data were also identified with TMM normalized and UQS normalized data. The genes with the largest |*log*_*2*_ *fold change*| values were well distributed by size (> 10 kb, 7.5%; 5–10 kb, 30%; 1–5 kb, 67.5%; < 1 kb, 7.5%) and ranged from 413 bp to ~ 28 kb.

### Biological relevance of differentially expressed genes

**Fig. 6:**
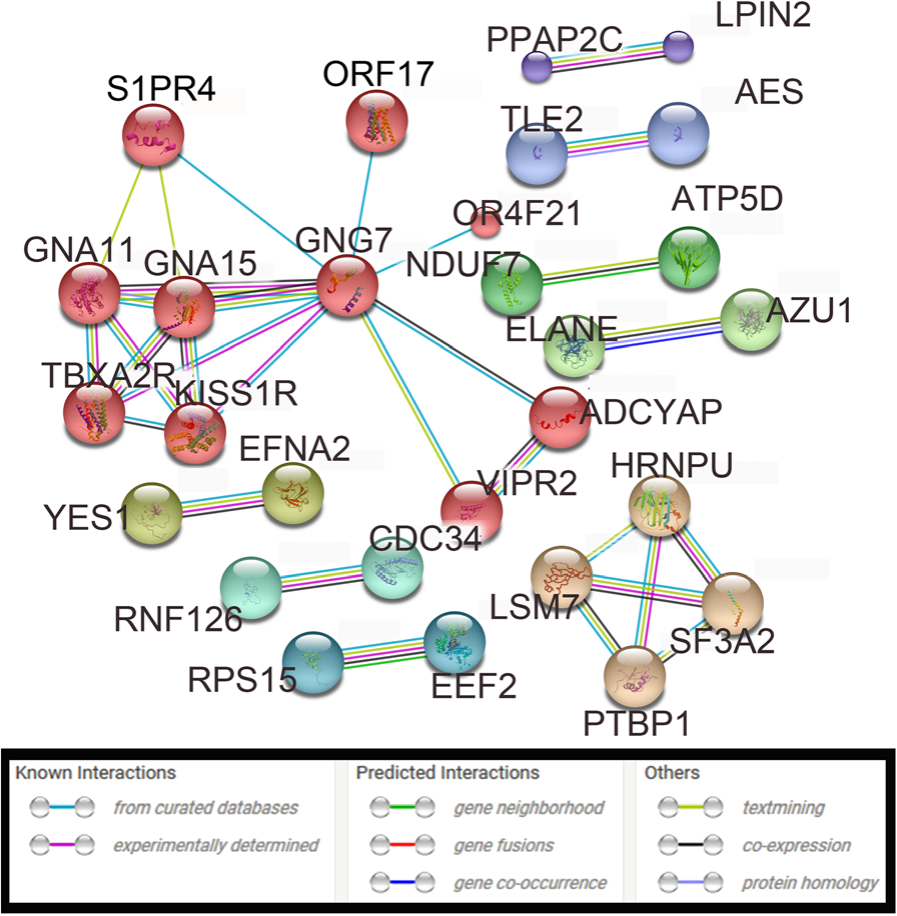
STRING analysis of protein networks. STRING identified 125 protein nodes within the 143 hNSC-upregulated genes. The nodes formed 9 distinct networks with 131 edges under the application of a high stringency filter. Protein nodes were mcl-clustered with an inflation parameter of three, and edges indicate various types of node interaction that are depicted by individual colors. Smaller protein nodes indicate a lack of 3D structural information about the individual protein

**Fig. 7:**
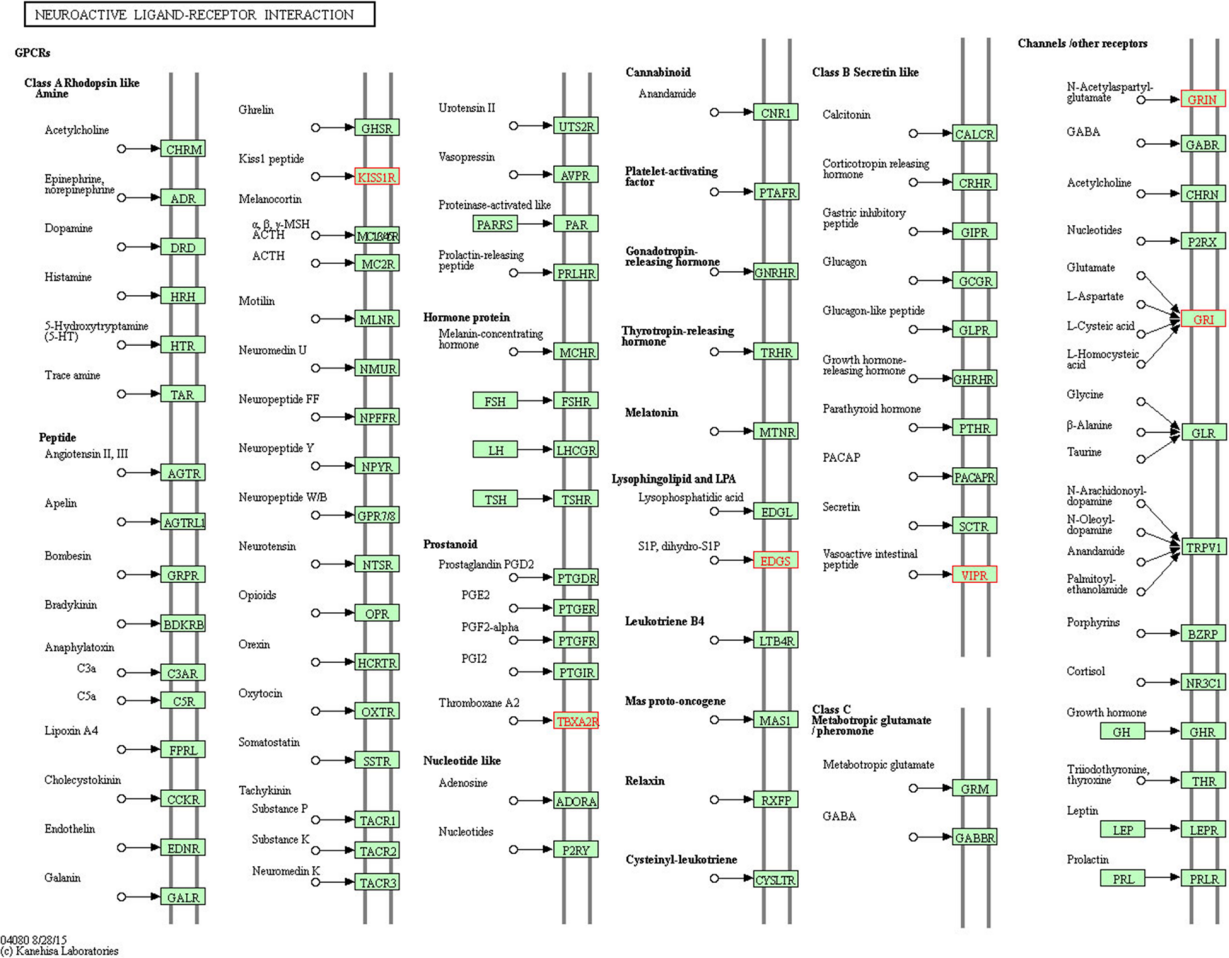
KEGG analysis of metabolic networks. KEGG analysis identified the most enriched metabolic networks for 143 hNSC-upregulated genes. Neurointeractive ligand-receptor interaction (depicted here) was one of the second most enriched networks for this gene list with five genes present in the network

The 143 differentially expressed genes that were identified with ANOVA analysis of TMM-normalized data were selected for downstream evaluation of potential emergent biological themes. All 143 genes were upregulated in neural stem cells by a margin of 1.4 to 1.5 times the expression level in normal human fetal-derived astrocytes (|*log*_*2*_ *fold change*| > 0.5*)*, and analyzed collectively. The online resource STRING identified 125 protein nodes, which formed 9 distinct networks with 31 edges under the application of a high stringency filter (Fig. 6, unconnected nodes removed) [31]. The average node degree was 0.496, and the local clustering coefficient average was 0.215. STRING revealed three significantly enriched GO terms in the human neural stem cell- upregulated genes: serine-type endopeptidase activity (8 genes), serine-type peptidase activity (7 genes), and phosphatidate phosphatase activity (3 genes). Analysis of the differentially expressed genes using KEGG (*Homo sapiens*) revealed that the most enriched metabolic pathways were “Metabolic pathways” (6 genes, not shown), “Neuroactive ligand-receptor interaction” (5 genes, Fig. 7), “cAMP signaling pathway” (5 genes, Additional file 1: Figure S1), PI3K-Akt signaling pathway (5 genes, Additional file 2: Figure S2) [32].

## Discussion

This study is a biological and technical assessment of RNA-Seq data from human cell lines that are used as experimental models for brain tissue. Reports of the potential for astroglia to transdifferentiate into neurons prompted the comparison of normal human fetal-derived astrocytes and human neural stem cell lines [16–20]. In our experiments, both cell types were cultured in identical conditions that are reported to initiate spontaneous differentiation of human neural stem cells. Phase contrast microscopy demonstrated that, although both cell lines propagate under identical spontaneous differentiation conditions, normal human fetal-derived astrocytes and human neural stem cells do not appear similar in size or shape (Fig. 1). Despite their morphological differences, a transcriptomic comparison of normal human fetal-derived astrocytes and human neural stem cells did not reveal significant differences in gene expression between the two cell lines according to our previously established threshold criteria for RNA-Seq analyses [16]. The transcriptomic similarity between human fetal-derived astrocytes and human neural stem cells supports the potential for human fetal-derived astrocyte transdifferentiation. This finding is congruent with previous investigations that have illuminated the plastic capacity of astrocytes (reviewed in [17]).

Results from this RNA-seq analysis stand in stark contrast to the results from microarray experiments that compare normal human fetal-derived astrocytes with human neural stem cells, where ~ 350 genes are reported to be upregulated by 5-fold in normal human fetal-derived astrocytes [33]. Although this discrepancy could be due to differences in technology (RNA-seq vs. microarray), or the number and type of replicate samples used for the statistical analyses, the differences in culture conditions are the most likely source of the different experimental outcomes. In the experiments by Malik et al. (2014), normal human fetal-derived astrocytes were cultured on uncoated tissue culture polystyrene in the media recommended by Lonza, and although the authors did not mention the substrate used for stem cell culture, the H9 derived human neural stem cells were cultured in the stem cell basal propagation media recommended by Gibco. In contrast, our data was acquired from normal human fetal-derived astrocytes and H9-derived human neural stem cells that were both cultured under identical conditions recommended by Gibco for spontaneous differentiation of human neural stem cells, including coating dishes with identical substrates (CellStart). Discrepancies between our outcomes and those of Malik et al. (2014) can be reconciled by the fact that human neural stem cells differentiate into astrocytes, oligodendrocytes, or neurons depending on the media and substrate selection.

The technical aspect of these experiments assesses variation that arises in the RNA-seq workflow, and compares the ability of different normalization methods to minimize unwanted variance. The many considerations that are necessary for the design of robust RNA-Seq experiments have been elegantly summarized by others [1, 34]. Critical factors include the sequencing platform, depth of coverage, and budget and availability constraints that limit the number of experimental samples. The technique selected for library preparation can be constrained by RNA yield and quality, which often reflect the nature of the experimental samples. The origin of the experimental sample also plays a role in the availability of biological or technical replicates for sequencing. The current study quantifies the contribution of a subset of experimental parameters to technical variance, and evaluates the effectiveness of normalization methods in minimizing this variance. Outcomes of our experiments are intended to inform researchers during the design of RNA-Seq experiments.

We quantified technical variation in an RNA-Seq comparison of two human neural cell lines based on cell lot, library preparation, and flow cell (Table 2). While flow cells differed in raw read count number (Table 3), only a small change in the distribution of the raw data was apparent when comparing across cell lots, library preparations, or flow cells (Fig. 2). Data normalization, which is intended to reduce technical noise and fit the data to similar distributions, was undertaken using RPKM, TMM, and UQS methods. RPKM normalization had a slight impact on the distribution of data as compared to the non-normalized (log_2_-transformed) samples, and marginally reduced the variance introduced by technical parameters of the experimental workflow by 1% (Figs. 3a, b, 4a, b, 5a-d). These findings are congruent with results from previous analyses [7, 22, 35, 36]. UQS normalization resulted in a similar data distribution across experimental samples, and reduced the technical variation in our data by 18% as compared with non-normalized samples (Figs. 3d, 4d, 5g, h). In contrast, TMM-normalization appeared to decrease the coherence of the data, and increased technical variance by 41% (Figs. 3c, 4c, 5e, f).

The increase in technical variance with TMM normalization that we observed stands in contrast to previous studies [36]. TMM normalization is favored in the literature for its ability to account for the large dynamic range of RNA-Seq data, minimize type I error, reduce variance, and retain the ability to detect differentially expressed genes [35–37]. In accordance with previous reports, TMM was the only normalization method that permitted the detection of differentially expressed genes in our analysis (FDR = 10%, |*log*_*2*_ *fold change*| > 0.5; *n* = 143; Table 4) [22, 35–37]. The increased technical variance that we observed following TMM normalization agrees with outcomes from a previous comparison of normalization methods based on bias and variance, where TMM normalization was not recommended, but normalization implemented by the DESeq and PoissonSeq packages for the R programming language performed well [38].

Concerns about replication and reproducibility in next generation sequencing analyses were the primary motivation for the technical component of this study [39]. The results from our principal variance component analysis support the recommendation for replicate library preparation made by the SEQC Consortium [40]. Moreover, these findings contribute to the body of literature that underscores the impact of normalization method on gene expression analyses [8, 22, 35–38, 41]. It is our hope that the quantification of technical variance presented in this manuscript empowers the decisions of investigators in the design of RNA-Seq experiments, and encourages the validation of normalization method before undertaking gene expression analyses.

## Conclusion

The transcriptomic comparison between human fetal-derived astrocytes and human neural stem cells revealed strong similarities between these two cell types. This finding adds to an expanding body of literature that highlights the neurogenic capacity of astrocytes, and warrants downstream investigations into their therapeutic potential. Principal variance component analysis of the 16 RNA-Seq data files revealed that library preparation introduced the greatest proportion of technical variance to the experiment. The three normalization methods differed in ability to reduce the technical variance introduced by different experimental parameters. We observed that the choice of normalization method affected our ability to detect differences in gene expression during comparative analysis of the neural and glial transcriptomes. Our results underscore the requirement for inclusion of replicate library preparations as part of RNA-Seq experimental design, and emphasize the importance of normalization method selection for differential expression analyses.

## Additional files

Additional file 1: **Figure S1** KEGG analysis of metabolic networks. KEGG analysis identified the most enriched metabolic networks for 143 hNSC-upregulated genes. “cAMP signaling pathway” (depicted here) was one of the second most enriched networks for this gene list with five genes present in the network. (TIF 9788 kb)

Additional file 2: **Figure S2** KEGG analysis of metabolic networks. KEGG analysis identified the most enriched metabolic networks for 143 hNSC-upregulated genes. “PI3K-Akt signaling pathway” (5 genes, (depicted here) was one of the second most enriched networks for this gene list with five genes present in the network. (TIF 9123 kb)

## Abbreviations

%: Percent
°C: Degrees Celsius
ANOVA: Analysis of variance
bp: Base pair
CCD: Charge-coupled device
cDNA: Complementary deoxyribonucleic acid
cm^2^: Square centimeter
CNS: Central nervous system
CO_2_: Carbon dioxide
CTS: Coding transcript sequence
DAVID: Database for Annotation, Visualization, and Integrated Discovery
DEG: Differentially expressed gene
DNA: Deoxyribonucleic acid
DPBS: Dulbecco’s modified phosphate buffered saline
G: Gravity
GEO: Gene Expression Omnibus
GFAP: Glial fibrillary acidic protein
GO: Gene Ontology Consortium
hESC: Human embryonic stem cell
hNSC: Human neural stem cell
HUGO: Human Gene Ontology
MIT: Massachusetts Institute of Technology
mRNA: Messenger RNA
NCBI: National center for biotechnology information
ng: Nanograms
NHA: Normal human astrocytes
NIH: National Institute of Health
NMSU: New Mexico State University
PBS: Phosphate buffered saline
PCR: Polymerase chain reaction
PE: Paired end
RIN: RNA integrity number
RNA: Ribonucleic acid
RNA-seq: RNA sequencing
RPKM: Reads per kilobase of exon per million reads mapped
RRID: Research resource identifiers
rRNA: Ribosomal RNA
SAM: Sequence alignment map
TMM: Trimmed means of M component
UCSC: University of California Santa Cruz
UQS: Upper quartile scaling
μg: Microgram
μL: Microliter
μm: Micrometer
