# Supplementary figures and images for: Expression analysis of RNA sequencing data from human neural and glial cell lines depends on technical replication and normalization methods
Source: BMC Bioinformatics. 2018 Nov 20;19(Suppl 14):412. doi: 10.1186/s12859-018-2382-0 (PMC6245503; doi:10.1186/s12859-018-2382-0)

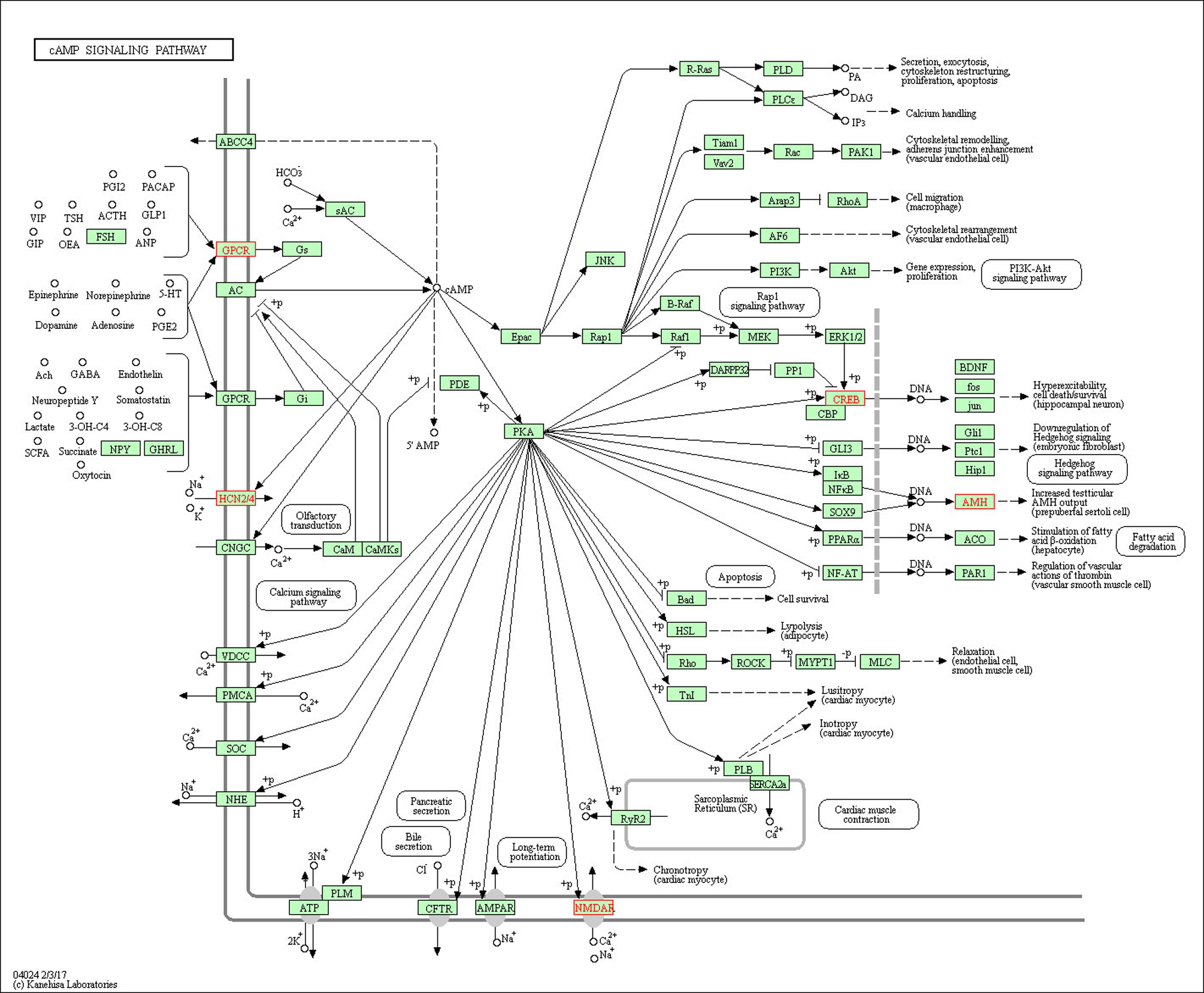

Supplement: Supplementary file 1 — Figure S1 KEGG analysis of metabolic networks. KEGG analysis identified the most enriched metabolic networks for 143 hNSC-upregulated genes. “cAMP signaling pathway” (depicted here) was one of the second most enriched networks for this gene list with five genes present in the network. (TIF 9788 kb) [file 12859_2018_2382_MOESM1_ESM.tif]

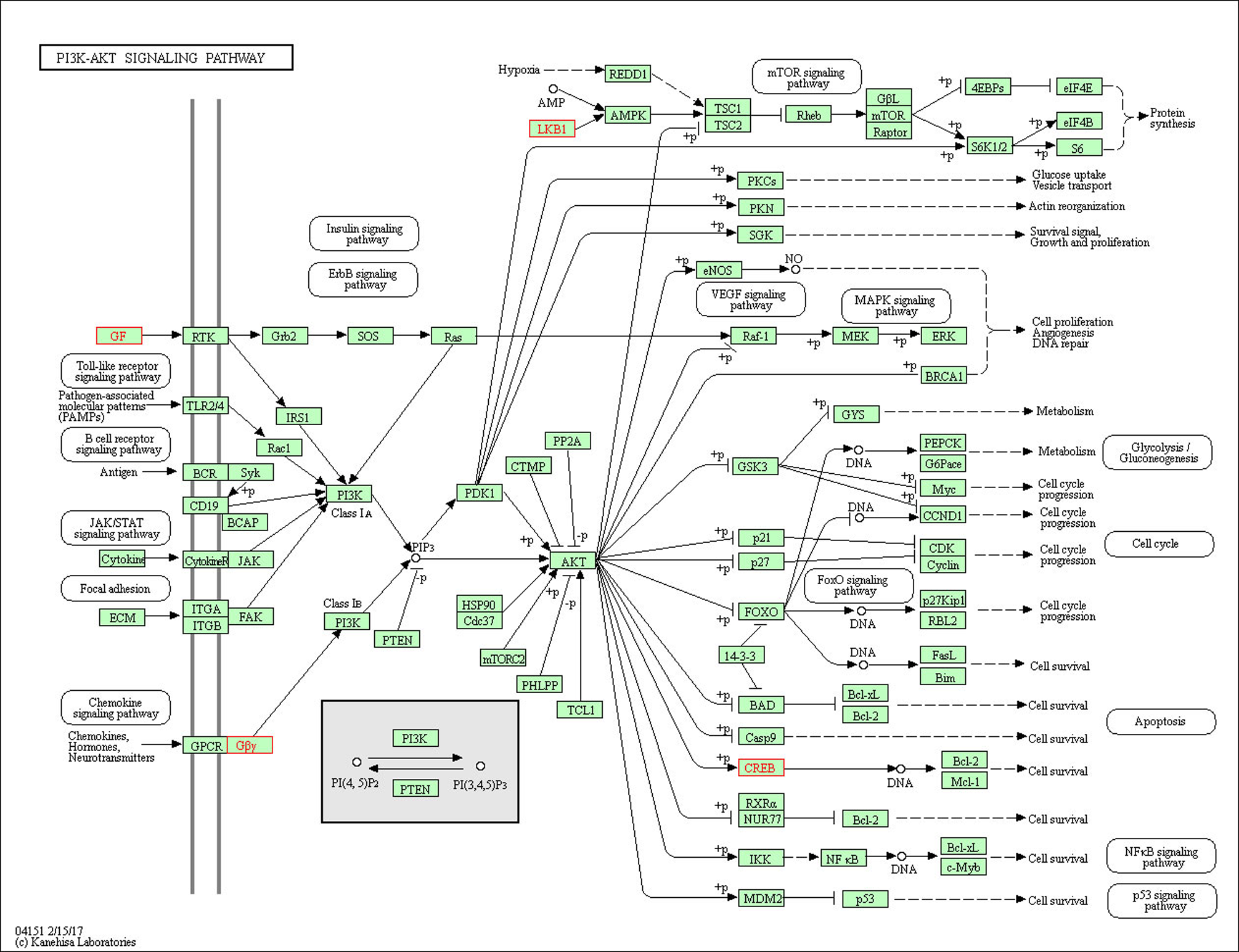

Supplement: Supplementary file 2 — Figure S2 KEGG analysis of metabolic networks. KEGG analysis identified the most enriched metabolic networks for 143 hNSC-upregulated genes. “PI3K-Akt signaling pathway” (5 genes, (depicted here) was one of the second most enriched networks for this gene list with five genes present in the network. (TIF 9123 kb) [file 12859_2018_2382_MOESM2_ESM.tif]
